# The Potential Role of Camel Milk in Alleviating Chronic Fatigue Syndrome in Mice: A Network Pharmacology and In Vivo Validation Study

**DOI:** 10.3390/foods15111861

**Published:** 2026-05-24

**Authors:** Hongman Li, Henigul Osman, Hongyan Zhang, He Chen, Nan Zheng, Yankun Zhao, Shiqi Zhang

**Affiliations:** 1 Laboratory of Quality and Safety Risk Assessment for Agro-Products of Ministry of Agriculture and Rural Affairs, Key Laboratory of Agro-Products Quality and Safety of Xinjiang, Urumqi 830091, China; lihong_m@163.com (H.L.); 13009693320@163.com (H.O.); zhanghongyan1978@xaas.ac.cn (H.Z.); chenhe_1971@163.com (H.C.); yankunzhao90@xaas.ac.cn (Y.Z.); 2 Institute of Quality Standards & Testing Technology for Agro-Products, Xinjiang Academy of Agricultural Sciences, Urumqi 830091, China; 3 State Key Laboratory of Animal Nutrition and Feeding, Institute of Animal Sciences, Chinese Academy of Agricultural Sciences, Beijing 100193, China; zhengnan@caas.cn

**Keywords:** camel milk, chronic fatigue syndrome, network pharmacology, functional food

## Abstract

Chronic fatigue syndrome/myalgic encephalomyelitis (CFS/ME) is a complex and debilitating disorder with limited treatment options. Camel milk (CM), known for its rich nutrients and anti-fatigue properties, may offer multi-target benefits for managing this condition. This study utilized an integrated approach combining metabolomics, network pharmacology, and animal experiments. CM metabolites were profiled and screened via ADME. Potential targets were predicted and intersected with CFS/ME-associated genes. Male BALB/c mice were subjected to chronic restraint and forced swimming to evaluate the effects of CM (1000 mg/kg) on behavioral, inflammatory, neuroendocrine, and metabolic parameters. CM administration significantly improved exhaustive swimming time and reduced immobility. It attenuated systemic inflammation (restored IL-10), normalized brain CREB and *DRD2*/*OPRM1* mRNA, and enhanced skeletal muscle AKT/GLUT4 expression and glycogen levels. Camel milk alleviates CFS/ME symptoms through the multi-component, multi-target regulation of neuroendocrine, inflammatory, and energy metabolism pathways. These preclinical findings suggest that CM may have potential as a supportive nutritional intervention for alleviating chronic fatigue, pending validation in human studies.

## 1. Introduction

Chronic fatigue syndrome/myalgic encephalomyelitis (CFS/ME) is a multi-system disease characterized by persistent or recurrent fatigue as its core symptom. It is often accompanied by sleep disturbances, cognitive decline, and muscle pain, which severely reduce patients’ quality of life [1,2]. Epidemiological surveys indicate that CFS/ME affects more than 10 million people worldwide. In Europe alone, the number of chronic fatigue cases reached 462,933 in 2018. Reports on its prevalence in the Chinese population are relatively limited; however, the prevalence among Chinese adolescents is 12%, significantly higher than the global average, with a high incidence in mental workers and students aged 10–18 years [3]. This condition represents an underlying pathological basis for impaired emotional regulation and cognitive dysfunction, posing a considerable public health challenge.

CFS/ME is a common chronic neurological disorder characterized by fatigue, but its exact etiology remains incompletely understood [4]. Current research suggests that its pathogenesis involves multiple factors, including genetic factors [5], psychological factors [6], virus-induced immune dysregulation [7], neuroendocrine disorders [8], and abnormal energy metabolism [9]. Severe fatigue reduces the activity level of CFS/ME patients by approximately 50% [10]. At present, there is no specific drug to alleviate CFS/ME. Existing treatments are mainly symptomatic, such as the use of selective serotonin reuptake inhibitors and non-steroidal anti-inflammatory drugs, but these show large individual differences and obvious side effects [11]. Therefore, exploring multi-target, low-risk intervention strategies has become an important research direction, and nutritional intervention is emerging as a promising field.

Camel milk is rich in high-quality proteins, essential fatty acids, and micronutrients and possesses antioxidant, anti-inflammatory, immunomodulatory, and anti-fatigue properties [12,13]. It may alleviate CFS/ME through multiple synergistic pathways: (1) metabolites in camel milk, such as tryptophan and γ-aminobutyric acid, improve the cAMP-AKT-CREB signaling pathway and upregulate brain-derived neurotrophic factor (BDNF), thereby maintaining cognitive function [14]; (2) camel milk provides nicotinamide, which improves the PI3K-AKT-GLUT4 signaling pathway, enhances mitochondrial energy metabolism efficiency, reduces lactate accumulation, and relieves exercise-induced fatigue [15]; (3) short-chain fatty acids and lactoferrin in camel milk inhibit the TLR4-NF-κB inflammatory pathway, reduce inflammatory cytokines, and decrease energy expenditure caused by inflammation [16]; (4) camel milk downregulates the expression of corticotropin-releasing hormone (CRH), pro-opiomelanocortin (POMC), and glucocorticoid receptor (NR3C1); inhibits excessive Hypothalamic–Pituitary–Adrenal (HPA) axis activation; and improves HPA axis dysfunction [17]. These properties provide a theoretical basis for using camel milk as an intervention for CFS/ME, but the molecular mechanisms and regulatory pathways still require empirical validation.

This study adopts a systematic approach integrating metabolomics, network pharmacology, and animal experiments to reveal the potential mechanism by which camel milk improves CFS/ME. First, metabolites in camel milk are identified using GC-MS and LC-MS, followed by ADME screening to obtain candidate active compounds. Second, network pharmacology is used to analyze the intersection between camel milk components and CFS/ME disease targets, and a “metabolite–target–pathway” network is constructed to identify key signaling pathways. Finally, a mouse model of CFS/ME is established to evaluate the effects of camel milk intervention on behavioral performance, metabolic indicators, and molecular expression in nerve and muscle tissues. Through network pharmacology, we aim to elucidate the potential targets and pathways through which camel milk components regulate CFS/ME, improve HPA axis function, inhibit inflammatory responses, and enhance energy metabolism, thereby systematically alleviating CFS/ME symptoms. This study provides a preclinical experimental basis for understanding the multi-target mechanisms of CM, which may inform future nutritional intervention strategies for CFS/ME.

## 2. Materials and Methods

### 2.1. Camel Milk Sample Collection

Raw camel milk was collected from 89 healthy, lactating Camelus bactrianus at a dairy station in Dabancheng District, Urumqi City, Xinjiang, China. Daily pooled samples (~300 mL) were prepared by homogenizing equal volumes from all individuals. Six independent biological replicates (one per day over six consecutive days) were stored in sterile, pre-chilled amber glass bottles and immediately frozen at −80 °C until analysis. Samples were thawed at 4 °C in the dark prior to metabolomic profiling.

### 2.2. Metabolite Profiling of Camel Milk

Metabolomic analysis was performed using gas chromatography–mass spectrometry (GC-MS) and liquid chromatography–mass spectrometry (LC-MS).

#### 2.2.1. GC-MS Analysis

A 100 μL aliquot of each sample was mixed with 400 μL of ice-cold methanol containing 0.5 mg/mL adonitol (internal standard), vortexed for 30 s, sonicated in an ice-water bath for 10 min, and centrifuged (12,000 rpm, 4 °C, 15 min). Then, 400 μL of the supernatant was dried under vacuum. For quality control (QC), 70 μL from each sample was pooled, dried, and derivatized with 30 μL of methoxyamine hydrochloride (20 mg/mL in pyridine) at 80 °C for 30 min, followed by 40 μL of BSTFA + 1% TMCS at 70 °C for 1.5 h. Before injection, 5 μL of fatty acid methyl ester standards (in chloroform) was added to QC samples. All samples were analyzed on an Agilent 7890B/5977B GC-MS system.

Metabolite profiling was conducted using a gas chromatography–mass spectrometry (GC-MS) system comprising an Agilent 7890B gas chromatograph coupled to a 5977B quadrupole mass spectrometer (Agilent Technologies, Santa Clara, CA, USA). Separation was achieved on a DB-5MS capillary column (30 m × 0.25 mm i.d., 0.25 μm film thickness). High-purity helium was used as the carrier gas at a constant flow rate of 1.0 mL/min. The inlet temperature was set at 280 °C, and 1 μL of the sample was injected in splitless mode. The oven temperature program was as follows: the initial temperature of 70 °C was held for 2 min, ramped to 180 °C at 10 °C/min, then increased to 280 °C at 5 °C/min, and finally held at 280 °C for 10 min.

The mass spectrometer was operated in electron ionization (EI) mode at 70 eV. The transfer line and ion source temperatures were maintained at 280 °C and 150 °C, respectively. Mass spectra were acquired in full-scan mode over a mass range of *m*/*z* 50–600 with a scan rate of 2.5 scans/s. Metabolites were identified by comparing their retention times and mass spectra with those of authentic standards and the NIST Mass Spectral Library (version 2017).

#### 2.2.2. LC-MS Analysis

Non-volatile metabolites were profiled on a Vanquish UHPLC system coupled to an Orbitrap Exploris 120 mass spectrometer (Thermo Fisher Scientific, Waltham, MA, USA). Samples (100 μL) were extracted with 400 μL of ice-cold methanol/acetonitrile (1:1, *v*/*v*) containing isotope-labeled internal standards (MSK-A2-1.2, Cambridge Isotope Laboratories). After vortexing (750 rpm, 5 min) and incubation (4 °C, 5 min), extracts were filtered (0.22 μm PVDF). Chromatographic separation was performed on a BEH Amide column (2.1 × 50 mm, 1.7 μm) with gradient elution (mobile phase A: water with 25 mM ammonium acetate and 25 mM ammonia; B: acetonitrile). The flow rate was 0.3 mL/min. MS detection used positive/negative switching mode (±3.8 kV), full-scan resolution of 60,000, and MS/MS resolution of 15,000. System stability was monitored by injecting a pooled QC sample every 10 runs.

### 2.3. Screening of Bioactive Metabolites

Raw data were processed using ProteoWizard (v3.0) and XCMS (v3.12). Features with >30% missing values or relative standard deviation (RSD) >25% in QC samples were removed. Probabilistic quotient normalization was applied. Compound annotation was performed using Compound Discoverer 3.3. Candidate bioactive compounds were selected via ADMET-AI (http://admet.ai.greenstonebio.com) based on oral bioavailability >30%, quantitative estimate of drug-likeness >0.5, and compliance with Lipinski’s Rule of Five. While these criteria are commonly used in drug discovery, they help prioritize camel milk metabolites that are more likely to exert systemic biological effects after oral administration.

### 2.4. Prediction of Potential Targets

Candidate compounds were submitted to TCMSP (v2.3), ChEMBL (v32; confidence ≥ 80, pChEMBL > 6), and STITCH (v6.0; Homo sapiens, score > 300). Predicted targets were merged, deduplicated, and standardized to official gene symbols.

### 2.5. Collection of Disease-Associated Targets

Targets associated with “Myalgic Encephalomyelitis” and “Chronic Fatigue Syndrome” were retrieved from GeneCards (v5.15), OMIM, and TTD.

### 2.6. Identification of Overlapping Targets

The intersection between compound-predicted targets and disease targets was identified using the R package VennDiagram (v1.6.20) and visualized as a Venn diagram.

### 2.7. Functional and Pathway Enrichment Analysis

Overlapping targets were subjected to Gene Ontology (GO) and KEGG pathway enrichment analyses using clusterProfiler (v4.6.2; Homo sapiens). Significance was defined as Benjamini–Hochberg-adjusted *p*-value < 0.05. Results were visualized using ggplot2 (v3.3.5).

### 2.8. Network Construction

Protein–protein interactions (PPIs) were analyzed using STRING (v11.0; confidence ≥ 0.4) and visualized in Cytoscape (v3.9.1). Hub targets were defined as those with degree values in the top 10%. A “camel milk metabolite–target–KEGG pathway” network was also constructed in Cytoscape.

### 2.9. Animal Study

#### 2.9.1. Ethical Approval

All animal procedures were performed in strict accordance with the recommendations in the Guide for the Care and Use of Laboratory Animals of the National Institutes of Health. The study protocol was approved by the Institutional Animal Care and Use Committee (IACUC) of Hangzhou HZT Bio-Tech Co., Ltd. (Hangzhou, China) (Approval No. IACUC/HTYJ-11934-01, 14 April 2025). All efforts were made to minimize suffering and reduce the number of animals used.

#### 2.9.2. Animals and Housing

Eighteen male BALB/c mice, weighing 18–22 g, were purchased from SPF (Suzhou) Biotechnology Co., Ltd. (Suzhou, China) (License No. SCXK (Su) 2022-0006). Upon arrival, the animals were acclimatized for one week prior to the experiment. They were housed in groups in polycarbonate cages with wood shavings as bedding, under standard laboratory conditions: a controlled temperature of 22 ± 2 °C, relative humidity of 50 ± 10%, and a 12 h light/dark cycle. Sterilized food and water were provided ad libitum throughout the study.

#### 2.9.3. Test Substances

The camel milk used in this study was provided as a lyophilized powder, which was reconstituted in purified water immediately before administration. Rhodiola capsules (Weihai ZiGuang Biotech (Weihai, China), batch No. A0524031) were used as the positive control drug and were prepared as an aqueous suspension.

#### 2.9.4. Experimental Design

The mice were randomly assigned to three experimental groups (*n* = 6 per group) using a random number table method to ensure allocation concealment:

Normal Control Group (Control): Received standard care without stress induction. Model Control Group (Model): Subjected to chronic fatigue syndrome (CFS) induction. Camel Milk Group (CM): Subjected to CFS induction and treated with camel milk (1000 mg/kg). The dosage of camel milk (1000 mg/kg) was selected based on the results of our preliminary experiments.

CFS Induction: The normal control group did not undergo the stress protocol. Mice in the model and CM groups underwent a combined stress protocol for 8 weeks [18,19], specified as follows:

Chronic Restraint: Mice were placed in well-ventilated restraint tubes for 3 h daily. Forced Swimming: Immediately following restraint, mice were forced to swim in water at 22 °C for 20 min.

Treatments (camel milk or vehicle) were administered daily via oral gavage at a volume of 10 mL/kg body weight. The body weight of each mouse was monitored weekly to adjust the dosage.

#### 2.9.5. Humane Endpoints and Euthanasia

Animals were monitored daily for signs of distress. Humane endpoints were predefined; any animal showing severe distress, significant weight loss (>20%), or inability to access food/water was excluded and euthanized. At the end of the 8-week period, all mice were euthanized via CO_2_ asphyxiation followed by cervical dislocation to ensure death, and tissues were harvested for analysis.

#### 2.9.6. Sample Collection

To minimize bias, the investigators conducting the behavioral tests and data analysis were blinded to the group allocation of the animals throughout the experiment. The primary outcome measures included behavioral changes (forced swim test, grip strength) and biochemical parameters.

#### 2.9.7. Behavioral Assessments

Locomotor activity and anxiety-like behavior were assessed using the open field test (5 min). Despair-like behavior was evaluated by the tail suspension test (6 min, immobility time recorded). Physical fatigue was measured by the forced swim test with a tail load (6% body weight, 25 °C; time to exhaustion recorded).

#### 2.9.8. Plasma Biomarker Analysis

Plasma levels of TNF-α, IL-10, and IL-17 were quantified using commercial ELISA kits (KJSBio) according to manufacturer instructions.

### 2.10. Related Biomarker in the Brain and Skeletal Muscle

#### 2.10.1. Quantitative Real-Time PCR

Total RNA was extracted from brain and skeletal muscle tissues using a TRIzol-based method. RNA purity (A260/A280 ≈ 1.8–2.0) was confirmed by Nanodrop 2000. First-strand cDNA was synthesized from 1 μg of RNA using SweScript All-in-One RT SuperMix. qPCR was performed on a Bio-Rad CFX Connect system with SYBR Green Master Mix. Relative expression of *BDNF*, *HTR1A*, *DRD2*, and *OPRM1* was calculated by the 2^−ΔΔCT^ method, normalized to *GAPDH*. Primer details are provided in Table 1.

#### 2.10.2. Western Blot Analysis

Total protein was extracted using RIPA lysis buffer supplemented with protease and phosphatase inhibitors. Protein concentration was determined by BCA assay. Equal amounts (5 μg) were separated by SDS-PAGE and transferred to PVDF membranes. Membranes were probed with primary antibodies against CREB, p-CREB (Ser133), AKT, and GAPDH (1:1000), followed by HRP-conjugated secondary antibodies (1:5000). Signals were detected using ECL reagents and imaged (SCG-W3000 PLUS).

### 2.11. Statistical Analysis

Data were analyzed using GraphPad Prism 10.6. One-way ANOVA with Tukey’s post hoc test was used for multi-group comparisons. Results are expressed as mean ± SEM. *p* < 0.05 was considered statistically significant.

## 3. Results

### 3.1. Metabolite Identification and Screening in Camel Milk

**Figure 1:**
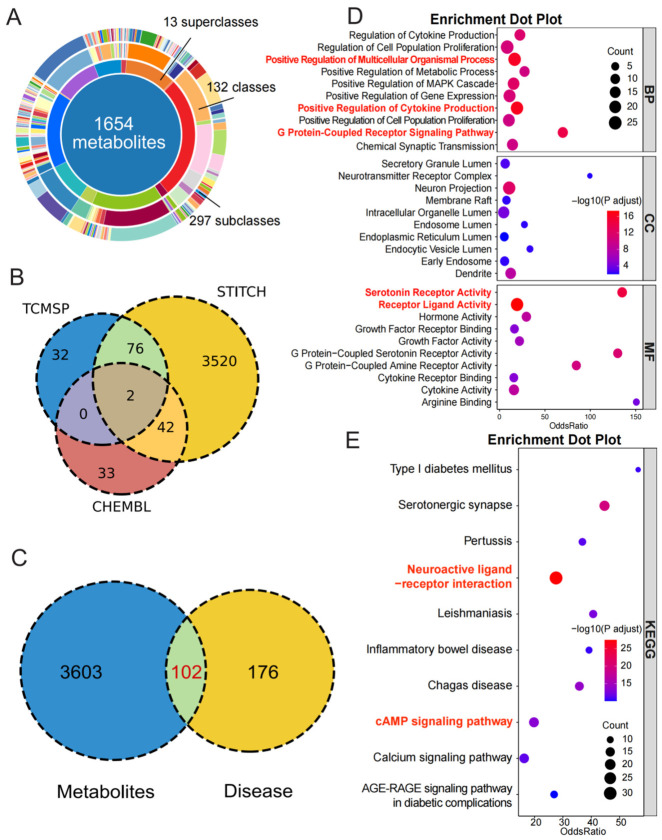
Chemical diversity and functional enrichment analysis of bioactive metabolites in camel milk. (**A**) Chemical classification of the 1654 metabolites identified in camel milk, spanning 13 superclasses. (**B**–**E**) Gene Ontology (GO) and Kyoto Encyclopedia of Genes and Genomes (KEGG) pathway enrichment analyses of the overlapping targets between camel milk metabolites and chronic fatigue syndrome. The dot plot shows the top enriched terms in Biological Process (BP), Cellular Component (CC), and Molecular Function (MF) categories, as well as KEGG pathways. The size of the dots represents the number of target genes, and the color intensity corresponds to −log10 (adjusted *p*-value).

A total of 1654 metabolites were identified in camel milk using GC-MS and LC-MS platforms. These metabolites were classified into 13 superclasses, 132 classes, and 297 subclasses (Figure 1A). To prioritize bioactive candidates for network pharmacology analysis, metabolites were filtered based on oral bioavailability (OB) > 30%, quantitative estimate of drug-likeness (QED) > 0.5, and compliance with Lipinski’s Rule of Five. This yielded 122 candidate compounds (Appendix A Table A1).

### 3.2. Overlapping Targets Between Camel Milk Compounds and CFS/ME

Through target prediction of 122 compounds using the TCMSP, ChEMBL, and STITCH databases, a total of 3705 unique potential targets were identified (Figure 1B). Disease-related targets for “Myalgic Encephalomyelitis/Chronic Fatigue Syndrome” (CFS/ME) were retrieved from the GeneCards, OMIM, and TTD databases, resulting in 278 disease-associated targets. Cross-analysis revealed 102 overlapping targets between camel milk compounds and CFS/ME (Figure 1C), involving 39 camel milk target metabolites (Appendix A Table A1, Top 39), suggesting their potential therapeutic value.

### 3.3. GO and KEGG Enrichment Analyses of Overlapping Targets

Gene Ontology (GO) enrichment analysis indicated that the 102 overlapping targets were significantly associated with biological processes including “regulation of cytokine production”, “positive regulation of cell population proliferation”, and “positive regulation of MAPK cascade” (adjusted *p* < 0.05). At the molecular function level, targets were enriched in “G protein-coupled receptor activity” and “neurotransmitter receptor complex”. Cellular component analysis highlighted enrichment in “neuronal projections” and “postsynaptic membrane” (Figure 1D).

KEGG pathway analysis revealed significant enrichment of overlapping targets in multiple signaling and metabolic pathways, including “serotonergic synapse”, “neuroactive ligand–receptor interaction”, “cAMP signaling pathway”, “calcium signaling pathway”, “AGE-RAGE signaling pathway in diabetic complications”, “inflammatory bowel disease”, and immune-related pathways such as “pertussis”, “leishmaniasis”, and “Chagas disease” (adjusted *p* < 0.05; Figure 1E).

### 3.4. PPI Network and “Metabolite–Target–Pathway” Network Construction

**Figure 2:**
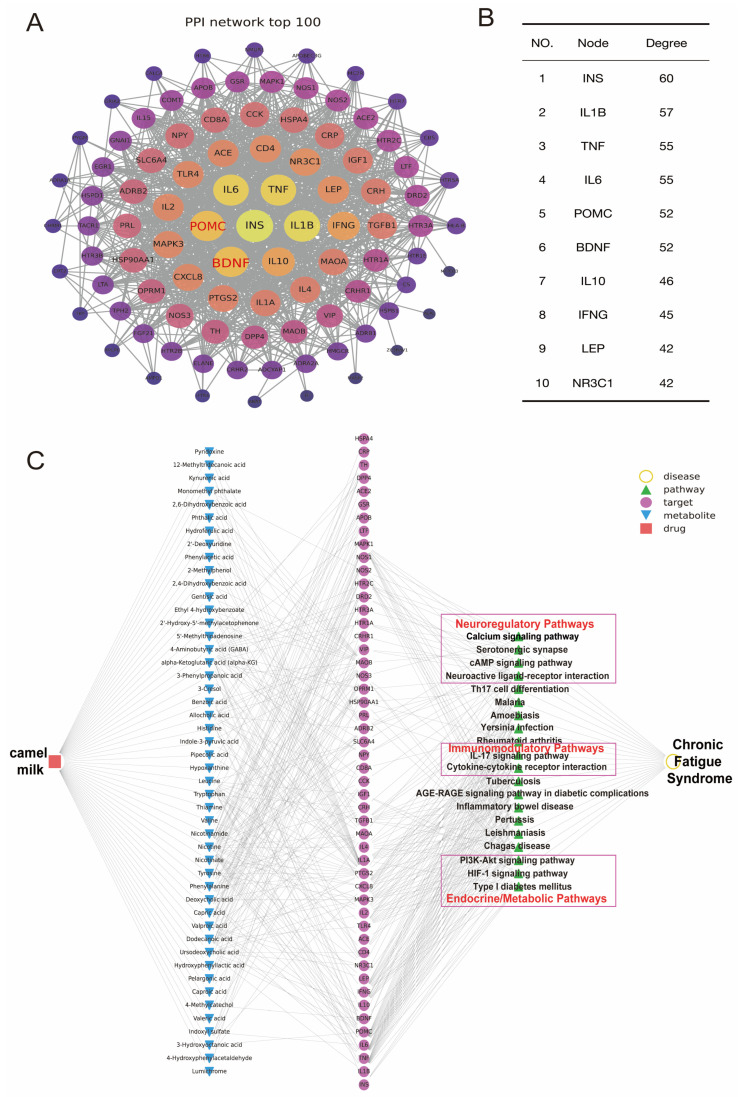
Network pharmacology analysis of camel milk metabolites against chronic fatigue syndrome. (**A**) Protein–protein interaction (PPI) network of the overlapping targets between camel milk metabolites and chronic fatigue syndrome (CFS). Node size and color intensity are proportional to the degree of connectivity. (**B**) The top 10 hub genes are labeled. (**C**) A multi-layered network integrating camel milk, its candidate bioactive metabolites, hub targets, and the corresponding enriched KEGG pathways, categorized into Neuroregulatory, Endocrine/Metabolic, and Immunomodulatory pathways.

Based on the 102 overlapping targets between camel milk and CFS/ME, a protein–protein interaction (PPI) network was constructed using the STRING database and visualized in Cytoscape, consisting of 102 nodes and multiple edges (Figure 2A). Topological analysis by degree centrality identified 10 hub targets with the top 10% degree values: INS, IL1B, TNF, IL6, POMC, BDNF, IL10, IFNG, LEP, and NR3C1 (Figure 2B). Systematic functional annotation of these hub targets (Figure 2C) revealed their involvement in three core biological domains: neuroendocrine regulation (POMC, BDNF, NR3C1, LEP), immune–inflammatory responses (IL1B, TNF, IL6, IL10, IFNG), and energy metabolism (INS, LEP). This classification aligns closely with the KEGG enrichment analysis, which highlighted pathways such as “serotonergic synapse”, “cAMP signaling pathway”, “AGE-RAGE signaling pathway”, and multiple immune-related pathways. Based on these findings, subsequent animal experiments were designed to validate the three predicted domains: namely, the effects of camel milk on (i) neuroendocrine function (brain BDNF, HTR1A, DRD2, OPRM1, and CREB signaling), (ii) immune–inflammatory responses (plasma TNF-α, IL-17, and IL-10), and (iii) energy metabolism (plasma lactate/urea, skeletal muscle AKT/GLUT4, glycogen, and ATP) in a murine CFS/ME model, thereby providing systematic experimental validation of the network pharmacology predictions.

### 3.5. Camel Milk Ameliorates Fatigue-Related Phenotypes in a Murine CFS/ME Model

To experimentally validate the three domains predicted by network pharmacology, a CFS/ME mouse model induced by chronic restraint combined with forced swimming was used.

Compared to the model group, the camel milk (CM) intervention significantly restored daily food intake (*p* < 0.05), bringing it to a level comparable to that in the control group. Body weight was significantly lower in the model group than in the control group (*p* < 0.05), whereas CM treatment reversed this reduction (*p* < 0.05 vs. model), with no significant difference between the CM and control groups.

In behavioral tests, the model group exhibited significantly shorter exhaustive swimming time under a 6% body weight load compared to the control group (*p* < 0.05). CM treatment significantly prolonged swimming endurance (*p* < 0.05 vs. model), restoring it to control levels. Similarly, immobility time in the tail suspension test was significantly increased in the model group (*p* < 0.05 vs. control), indicating despair-like behavior; CM administration significantly reduced immobility time (*p* < 0.05 vs. model).

**Figure 3:**
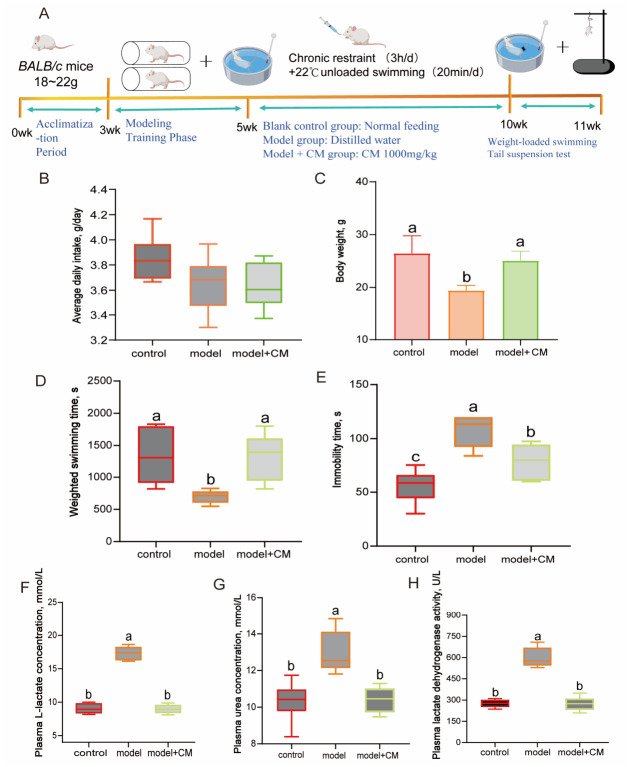
Camel milk supplementation alleviates physical fatigue and improves exercise tolerance in a mouse (**A**) model of chronic fatigue syndrome. (**A**) Experimental timeline and effects of camel milk (CM, 1000 mg/kg) on physical performance and metabolic markers in a CFS mouse model. Mice were subjected to chronic restraint and forced swimming for 8 weeks to induce CFS-like symptoms. (**B**) Average daily food intake. (**C**) Body weight progression over the period. (**D**) Weighted swimming time to exhaustion. (**E**) Immobility time in the tail suspension test. (**F**) Plasma L-lactate concentration. (**G**) Plasma urea concentration. (**H**) Plasma lactate dehydrogenase activity. Data are presented as mean ± SEM (n = 6). Different letters (a, b, c) above bars indicate statistically significant differences between groups (*p* < 0.05) by one-way ANOVA with Tukey’s post hoc test.

Biochemical analysis showed that plasma L-lactate concentration was significantly elevated in the model group compared to the control group (*p* < 0.05), and CM treatment significantly lowered lactate levels (*p* < 0.05 vs. model). Plasma urea concentration followed a similar pattern: increased in the model (*p* < 0.05 vs. control) and reduced by CM (*p* < 0.05 vs. model). Additionally, lactate dehydrogenase activity was significantly higher in the model group (*p* < 0.05 vs. control) and was normalized by CM intervention (*p* < 0.05 vs. model; no significant difference vs. control) (Figure 3). These results align with the predicted involvement of energy metabolism dysregulation in CFS/ME.

### 3.6. Camel Milk Modulates Systemic Inflammatory and Immune Responses

**Figure 4:**
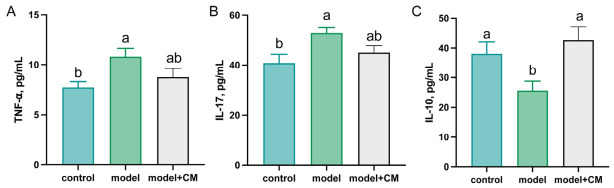
Effects of camel milk (CM, 1000 mg/kg) on plasma levels of key inflammatory and immune cytokines in a CFS mouse model. (**A**) Plasma tumor necrosis factor-alpha (TNF-α) concentration. (**B**) Plasma interleukin-10 (IL-10) concentration. (**C**) Plasma interleukin-17 (IL-17) concentration. Data are presented as mean ± SEM (n = 6). Different letters (a, b) above bars indicate statistically significant differences between groups (*p* < 0.05) by one-way ANOVA with Tukey’s post hoc test.

Chronic stress induced a significant pro-inflammatory state, as evidenced by elevated plasma TNF-α and IL-17 (model vs. control, *p* < 0.05, Figure 4A,B), while the anti-inflammatory cytokine IL-10 showed a significant decrease (model vs. control, *p* < 0.05, Figure 4C). Treatment with camel milk restored IL-10 levels to those observed in the control group (*p* < 0.05). For TNF-α and IL-17, although CM treatment showed a trend toward reduction, the differences between the model and CM groups did not reach statistical significance (*p* > 0.05). These findings directly validate the immune-related KEGG pathways and the hub targets (IL10) identified in network pharmacology.

### 3.7. Camel Milk Restores Neuroendocrine Regulation and Neuroplasticity in the Brain

**Figure 5:**
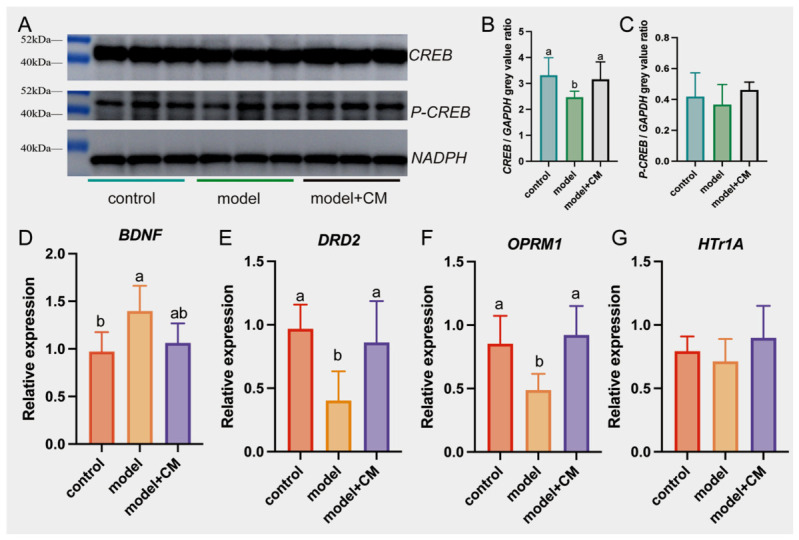
Camel milk regulates the expression of key neuroregulatory genes and proteins in the brain of a mouse model of chronic fatigue syndrome. (**A**) Western blot analysis of CREB and its phosphorylated form (p-CREB) in brain tissue. (**B**,**C**) Quantification of p-CREB/GAPDH and CREB/GAPDH protein ratios. (**D**–**G**) Relative mRNA expression levels of BDNF, DRD2, OPRM1, and HTR1A in brain tissue, as determined by qRT-PCR. Data are presented as mean ± SEM (n = 6). Different letters (a, b) above bars indicate statistically significant differences between groups (*p* < 0.05) by one-way ANOVA with Tukey’s post hoc test.

As shown in Figure 5A–C, chronic stress (model group) significantly reduced the total CREB/GAPDH ratio compared with the control group (*p* < 0.05). In contrast, the p-CREB/GAPDH ratio did not differ significantly among the three groups. Oral administration of CM markedly elevated the total CREB/GAPDH ratio to a level comparable with that of the control group (*p* < 0.05 vs. model), while the p-CREB/GAPDH ratio remained unchanged. These results indicate that in this CFS/ME model, the deficit lies in total CREB protein expression rather than its phosphorylation status, and camel milk restores total CREB without affecting p-CREB levels.

Quantitative RT-PCR analysis (Figure 5D–G) revealed that, compared with the control group, the mRNA expression level of *BDNF* in the brain was significantly upregulated in the model group (*p* < 0.05), whereas the expression levels of DRD2 and *OPRM1* were significantly downregulated (*p* < 0.05). These opposing directional changes—elevated *BDNF* alongside reduced *DRD2*/*OPRM1*—suggest a maladaptive compensatory response to chronic stress rather than a simple neurotrophic deficit. CM treatment effectively reversed these changes: the expression levels of *BDNF*, *DRD2* and *OPRM1* were similar to those in the control group (*p* > 0.05 compared with the control group). No significant differences were observed between the CM group and the control group for any of the genes tested (*p* > 0.05). Thus, camel milk normalizes directionally distinct molecular abnormalities in the brain, restoring both the abnormally elevated BDNF and the suppressed DRD2/OPRM1 to control levels. These results directly validate the top KEGG pathways predicted by network pharmacology, including “neuroactive ligand–receptor interaction” (DRD2, OPRM1) and “cAMP signaling pathway” (CREB). The restoration of total CREB further supports the involvement of neuroplasticity mechanisms.

### 3.8. Camel Milk Improves Skeletal Muscle Energy Metabolism

**Figure 6:**
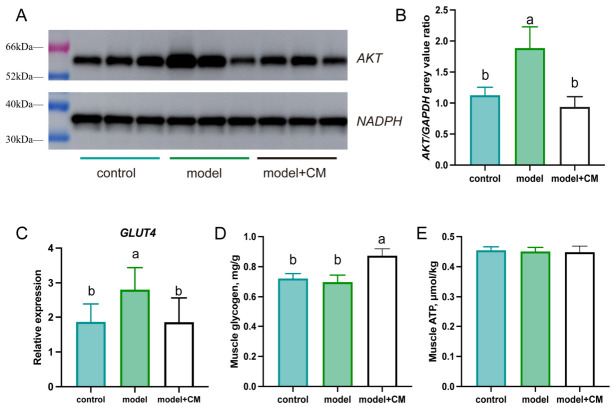
Effects of camel milk on skeletal muscle energy metabolism in a CFS mouse model. (**A**) Western blot analysis of AKT protein in skeletal muscle. (**B**) Quantification of AKT/GAPDH protein ratio. (**C**) Relative mRNA expression of the glucose transporter *GLUT4*. (**D**) Muscle glycogen content. (**E**) Muscle ATP content. Data are presented as mean ± SEM (n = 6). Different letters (a, b) above bars indicate statistically significant differences between groups (*p* < 0.05) by one-way ANOVA with Tukey’s post hoc test.

Given the predicted involvement of energy metabolism-related hub targets (INS, LEP) and the observed changes in plasma lactate/urea, we further examined insulin/AKT signaling and glucose utilization in skeletal muscle. As shown in Figure 6A,B, the AKT/GAPDH protein ratio was significantly higher in the model group than in the control group (*p* < 0.05). Oral administration of CM markedly increased the AKT/GAPDH ratio to a level comparable to that of the control group (*p* < 0.05 vs. model). Consistent with these changes, the relative mRNA expression of the glucose transporter *GLUT4* in skeletal muscle (Figure 6C) was significantly upregulated in the model group compared with the control group (*p* < 0.05), and CM treatment effectively restored *GLUT4* expression to control levels (*p* < 0.05 vs. model). Furthermore, CM administration significantly elevated muscle glycogen content (Figure 6D, *p* < 0.05 vs. model). No significant differences were observed between the CM-treated group and control groups for muscle ATP levels (Figure 6E).

## 4. Discussion

In this study, we systematically investigated the therapeutic potential of camel milk (CM) in a murine model of myalgic encephalomyelitis/chronic fatigue syndrome (CFS/ME) by integrating metabolomics, network pharmacology, and experimental validation. A total of 1654 metabolites were identified in CM, of which 39 candidate compounds met the ADME/drug-likeness criteria and targeted 102 overlapping genes associated with CFS/ME. Subsequent in vivo experiments demonstrated that CM intervention significantly ameliorated fatigue-related phenotypes, suppressed systemic inflammation, restored neuroendocrine function, and improved skeletal muscle energy metabolism. These findings provide evidence that CM acts through a multi-target “neuro-immune-metabolic network” to achieve systematic intervention in CFS/ME, consistent with the principle that natural products exert therapeutic effects via synergistic actions of multiple metabolites [12,13].

### 4.1. Network Pharmacology Reveals Multi-Target Mechanisms of CM in CFS/ME

The network pharmacology analysis revealed that the 102 overlapping targets were significantly enriched in biological processes including cytokine production, neurotransmitter receptor complex, and G protein-coupled receptor signaling, as well as in key pathways such as neuroactive ligand–receptor interaction, cAMP signaling, and PI3K-AKT signaling. Topological analysis identified 10 hub targets (INS, IL1B, TNF, IL6, POMC, BDNF, IL10, IFNG, LEP, NR3C1), which clustered into three core functional domains: neuroendocrine regulation (POMC, BDNF, NR3C1, LEP), immune–inflammatory responses (IL1B, TNF, IL6, IL10, IFNG), and energy metabolism (INS, LEP). This multi-target profile aligns well with the multifactorial pathogenesis and multi-system involvement of CFS/ME [20,21].

### 4.2. CM Suppresses Systemic Inflammation by Modulating Cytokine Networks

Chronic low-grade inflammation is widely recognized as a core feature of CFS/ME. Our experimental results showed that CM treatment significantly normalized the anti-inflammatory cytokine IL-10 in CFS/ME model mice. These findings are consistent with clinical observations that elevated inflammatory cytokines (e.g., IL-6, TNF-α) are positively correlated with fatigue severity in CFS/ME patients, and exaggerated immune signaling may contribute to both muscle and systemic fatigue [22]. Network enrichment analysis indicated that CM metabolites can regulate inflammatory mediators such as TLR, IL-6, and TNF-α, with short-chain fatty acids and lactoferrin proposed as candidate factors that inhibit the TLR4–NF-κB pathway and downregulate pro-inflammatory cytokines [16,23]. CM has the potential to inhibit IL-17; although the data from this study did not reveal a significant difference, there was a clear trend towards inhibition. Given that Th17 dysregulation is one of the causes of neuroinflammation in CFS/ME, camels’ milk administered at different doses may yield better results in future studies [24,25]. Furthermore, the restoration of IL-10 indicates that CM restores immune homeostasis, rather than broadly suppressing immune function [26,27].

### 4.3. Camel Milk Restores Neuroendocrine Function and Neuroplasticity via Multi-Directional Normalization

Our network pharmacology analysis highlighted significant enrichment of overlapping targets in pathways including “neuroactive ligand–receptor interaction” (e.g., DRD2, OPRM1) and “cAMP signaling pathway” (e.g., CREB). The subsequent in vivo results provided direct experimental validation of these predictions while also revealing an unexpected but important pattern regarding neurotrophic regulation [27,28].

Chronic stress in CFS/ME model mice led to a significant reduction in total CREB protein expression, which was fully reversed by camel milk (CM) treatment. In contrast, the ratio of phosphorylated CREB (p-CREB) to GAPDH did not differ significantly among the three groups. This finding is noteworthy because most studies focus on p-CREB at Ser133 as the surrogate for transcriptional activity. Our data suggest that in this specific CFS/ME model, the limiting factor may be the total CREB protein pool rather than its phosphorylation status. The restoration of total CREB by CM indicates that the intervention supports CREB synthesis or stability, thereby maintaining the capacity for cAMP-responsive gene expression, which is essential for synaptic plasticity and neuroendocrine adaptation [29,30].

We observed a significant upregulation of BDNF mRNA in the model group, which was reduced by CM treatment to control levels. At first glance, this appears contrary to the expected neuroprotective role of BDNF. However, accumulating evidence suggests that chronic stress can induce a compensatory but maladaptive increase in BDNF in certain brain regions, which may contribute to allostatic load rather than genuine resilience [31]. The normalization of BDNF by CM therefore likely reflects restoration of neurotrophic homeostasis, not a simple augmentation. In parallel, DRD2 (dopamine receptor D2) and OPRM1 (mu-opioid receptor) were significantly downregulated in model mice—a change consistent with the anhedonia, fatigue, and pain symptoms characteristic of CFS/ME [32]. CM treatment effectively restored their expression to control levels, directly validating the predicted “neuroactive ligand–receptor interaction” pathway.

Together, these results demonstrate that CM does not uniformly activate or suppress a single signaling axis; rather, it normalizes directionally distinct abnormalities depending on the molecular target. The concurrent restoration of total CREB, DRD2, and OPRM1, alongside the correction of aberrantly elevated BDNF, supports the view that CM acts as a homeostatic modulator of neuroendocrine and neuroplasticity pathways. These effects provide a mechanistic basis for the observed behavioral improvements (reduced immobility in the tail suspension test and prolonged swimming endurance) and align with the multi-target nature of CFS/ME pathophysiology.

### 4.4. Camel Milk Improves Skeletal Muscle Energy Metabolism via the AKT-GLUT4 Axis

Post-exertional malaise and energy depletion are debilitating aspects of CFS/ME. Our results showed that CM treatment significantly prolonged exhaustive swimming time and reduced plasma lactate and urea levels. Mechanistically, we observed significant downregulation of AKT protein and *GLUT4* mRNA in the skeletal muscle of model mice, which was reversed by CM treatment. The PI3K-AKT signaling pathway is central to insulin signaling and glucose uptake [33]. Impaired AKT signaling leads to reduced GLUT4 translocation, limiting glucose entry into muscle cells and forcing anaerobic glycolysis, resulting in lactate accumulation—a hallmark of CFS/ME [34]. Inflammatory cytokines such as IL-6 and TNF-α can dysregulate AKT/PKB signaling, decreasing AS160 phosphorylation and blocking GLUT4 vesicle translocation [35]. Our results are consistent with this mechanism, as CM simultaneously reduced systemic inflammation and restored AKT-GLUT4 signaling. Furthermore, CM restored muscle glycogen content, indicating improved energy status.

It should be noted that while BCAA-activated mTORC1 can promote mitochondrial biogenesis, some mitochondrial disease models show that mTOR inhibition (e.g., with rapamycin) delays disease progression, increases autophagy/mitophagy, and restores metabolic profiles [36]. Long-term excessive mTOR activation may inhibit autophagy, leading to accumulation of defective mitochondria [37]. Therefore, future studies using CM should monitor tissue-specific mTOR activity, mitochondrial membrane potential, ROS, and lactate levels.

### 4.5. Integrated Mechanisms and Therapeutic Implications

Collectively, our findings demonstrate that CM alleviates CFS/ME through coordinated modulation of three interconnected domains: (1) suppression of systemic and neuroinflammation via downregulation of TNF-α and IL-17 and upregulation of IL-10; (2) restoration of neuroendocrine function and neuroplasticity via upregulation of CREB, HTR1A, DRD2, and OPRM1 and downregulation of BDNF and NR3C1; and (3) improvement of skeletal muscle energy metabolism via activation of the AKT-GLUT4 axis, replenishment of glycogen and ATP, and reduction in lactate [38,39]. The multi-target nature of CM is particularly advantageous for a systemic disorder like CFS/ME, where single-target interventions have shown limited success [38]. This study provides a robust paradigm integrating network pharmacology with experimental validation to decipher the “multi-component, multi-target, multi-pathway” mechanisms of natural products [39]. Nevertheless, several methodological and interpretative limitations should be carefully considered, as discussed below.

### 4.6. Limitations and Future Perspectives

Several limitations of this study should be acknowledged, which also inform key directions for future research.

First, the animal model and experimental design. The CFS/ME model was induced by chronic restraint combined with forced swimming, which captures stress-related fatigue but does not fully replicate the heterogeneity, post-exertional malaise, or viral/immune triggers seen in human CFS/ME. Moreover, only male BALB/c mice were used, and the sample size was limited (n = 6 per group). While the preliminary data provide clear proof-of-concept, the exclusion of female animals prevents generalization of the findings to the entire population, given known sex differences in immune and neuroendocrine responses. Future studies must include both sexes with larger cohorts (minimum n = 10 per group) to improve statistical power and enable subgroup analyses.

Second, the sample size (n = 6) and single-dose design. Although statistically significant differences were observed for several endpoints, the small group size increases the risk of type II errors and limits the robustness of some comparisons (e.g., IL-17, OPRM1). In addition, only one dose of camel milk (1000 mg/kg) was tested. Dose–response studies (e.g., 250, 500, 1000, 2000 mg/kg) are necessary to establish the optimal therapeutic window and to determine whether certain effects reach significance at higher or lower doses.

Third, the use of human target databases for network pharmacology. Candidate targets were predicted using human-derived databases (TCMSP, ChEMBL, STITCH), while the experimental validation was performed in mice. Although many genes and pathways are conserved between species, cross-species discrepancies exist. Future work should incorporate mouse-specific target prediction or perform comparative cross-species analyses to strengthen translational relevance.

Fourth, the ADME/drug-likeness criteria (OB > 30%, QED > 0.5). These criteria were originally developed for pharmaceutical compounds. Their application to food-derived metabolites may introduce bias, as many nutritional components exert local effects in the gut or act via microbiome-derived metabolites rather than requiring systemic bioavailability. Furthermore, the specific bioactive metabolites responsible for the observed effects remain unidentified. While 39 candidate compounds were prioritized, the study did not isolate or test individual metabolites. Future research should employ activity-guided fractionation, metabolomics-driven correlation analysis, and validation using pure compounds (e.g., nicotinamide, tryptophan, short-chain fatty acids) to pinpoint key effectors.

Fifth, mechanistic depth. The study measured total CREB and p-CREB but did not assess upstream or downstream components of the cAMP signaling pathway. Similarly, while *GLUT4* mRNA was quantified, GLUT4 protein translocation and sarcolemmal localization were not examined. Future studies should include immunohistochemistry or subcellular fractionation to assess GLUT4 membrane trafficking. In addition, the effect of CM on brain GLUT4 and its relationship with cognitive function merits further investigation, including assessments of brain glucose uptake, ATP levels, and hippocampal long-term potentiation [40].

Sixth, translational relevance. The murine CFS/ME model used in this study reflects stress-induced fatigue but does not capture the full clinical complexity of human CFS/ME, including viral triggers, immune dysregulation, and long-term disability. While the findings suggest that camel milk may serve as a supportive nutritional intervention, claims regarding its efficacy as a “functional food for managing CFS” should be interpreted with caution. Human clinical trials are urgently needed to evaluate the safety, tolerability, and efficacy of camel milk in CFS/ME patients, with attention to dose, treatment duration, and patient stratification (e.g., by sex, disease severity, or inflammatory status).

Finally, the lack of direct binding validation of key metabolites to their predicted targets. Techniques such as surface plasmon resonance, cellular thermal shift assays, or molecular docking followed by mutagenesis would provide direct evidence for metabolite–target interactions.

In summary, while this study provides a robust and innovative framework integrating multi-omics with experimental validation, the above limitations highlight the preliminary, hypothesis-generating nature of the findings. Future studies addressing these issues will be essential to establish camel milk as an evidence-based nutritional strategy for CFS/ME.

## 5. Conclusions

This study integrated metabolomics, network pharmacology, and animal experiments to investigate the mechanisms of camel milk (CM) against CFS/ME. From 1654 metabolites identified in CM, 39 candidate compounds were selected after ADME screening, targeting 102 overlapping CFS/ME-related genes. Network pharmacology revealed enrichment in neuroendocrine, immune-inflammatory, and energy metabolism pathways.

In male BALB/c mice used as a CFS/ME model, CM intervention significantly improved fatigue-related behaviors (prolonged exhaustive swimming time, reduced immobility time in tail suspension test), suppressed systemic inflammation (restored IL-10), restored total CREB protein expression in the brain, corrected aberrant brain mRNA expression of *DRD2*/*OPRM1* (reduced in model group and restored by CM), improved skeletal muscle glycogen level, and decreased plasma lactate and urea. These findings suggest that camel milk has the potential to alleviate CFS/ME by modulating inflammation, neuroplasticity and energy metabolism via multiple targets. Future studies should identify key bioactive components and validate target necessity via genetic approaches.

## Figures and Tables

**Table 1:** Primer sequences.

Target Gene	NCBI RefSeq ID	Primer Name	Sequence (5′-3′)	Product Length (bp)	Annealing Temperature (Tm, °C)
*GAPDH*	NM_008084.2	M-GAPDH-S	CCTCGTCCCGTAGACAAAATG	133	60
		M-GAPDH-A	TGAGGTCAATGAAGGGGTCGT		
*GLUT4*	NM_001359114.1	M-GLUT4-S	GCTGAAGGATGAGAAACGGAAGT	259	60
		M-GLUT4-A	TTCTACTAAGAGCACCGAGACCAA		
*BDNF*	NM_001048139.1	M-BDNF-S	TATTAGCGAGTGGGTCACAGCG	213	60
		M-BDNF-A	TACGATTGGGTAGTTCGGCATT		
*Pomc(1)*	NM_001278581.1	M-Pomc(1)-S	TTCCTGGCAACGGAGATGAA	170	60
		M-Pomc(1)-A	ACTCGGCTCTGGACTGCCAT		
*Htr1a*	NM_008308.4	M-Htr1a-S	ACTCCACTTTCGGCGCTTTC	181	60
		M-Htr1a-A	GGCTGACCATTCAGGCTCTTC		
*Drd2*	NM_001410193.1	M-Drd2-S	CAGCAGAAGGAGAAGAAAGCCAC	146	60
		M-Drd2-A	CATGTGAAGGCGCTGTAGAGGA		
*Oprm1*	NM_001039652.2	M-Oprm1-S	ACTTCCTGGTCATGTATGTGATTGT	218	60
		M-Oprm1-A	GAGGGTGAAGATACTGGTGAACAT		
*NR3C1*	NM_001361209.1	M-NR3C1-S	GACTCAGCATGGAGAATTATGACCA	96	60
		M-NR3C1-A	TCTGAATCCTGGTATCGCCTTT		

## Data Availability

The original contributions presented in this study are included in the article. Further inquiries can be directed to the corresponding author.

## Abbreviations

The following abbreviations are used in this manuscript:

AKT	Protein Kinase B
BDNF	Brain-Derived Neurotrophic Factor
cAMP	Cyclic Adenosine Monophosphate
CFS/ME	Chronic Fatigue Syndrome/Myalgic Encephalomyelitis
ChEMBL	ChEMBL database
CM	Camel Milk
CREB	cAMP Response Element-Binding Protein
CRH	Corticotropin-Releasing Hormone
DRD2	Dopamine Receptor D2
GC-MS	Gas Chromatography-Mass Spectrometry
GLUT4	Glucose Transporter Type 4
HPA	Hypothalamic–Pituitary–Adrenal (axis)
HRP	Horseradish Peroxidase
HTR1A	5-Hydroxytryptamine Receptor 1A
IFNG	Interferon-Gamma
IL6	Interleukin-6
IL10	Interleukin-10
INS	Insulin
KEGG	Kyoto Encyclopedia of Genes and Genomes
NF-κB	Nuclear Factor Kappa-B
NR3C1	Glucocorticoid Receptor
OPRM1	Mu-Opioid Receptor
PI3K	Phosphoinositide 3-Kinase
POMC	Pro-Opiomelanocortin
PPI	Protein–Protein Interaction
TCMSP	Traditional Chinese Medicine Systems Pharmacology
TLR4	Toll-Like Receptor 4
TNF	Tumor Necrosis Factor
TTD	Therapeutic Target Database
UHPLC	Ultra-High Performance Liquid Chromatography

## Appendix A

**Table A1:** Selection criteria for 122 metabolites based on oral bioavailability, drug-likeness, and Lipinski’s Rule of Five.

No.	Substance Name	Mass-to-Charge Ratio	Molecular Formula	CAS Number	Human Metabolome Database	Target Metabolites
1	Xanthoxylin	195.0666	C_7_H_8_O_2_	90-24-4	HMDB0029645	YES
2	3-Methylcatechol	123.0454	C_7_H_8_O_2_	488-17-5	HMDB0301753	YES
3	3-methylolphenol	123.0454	C_7_H_8_O_2_	620-24-6	HMDB0059712	YES
4	4-Methylcatechol	123.0454	C_7_H_8_O_2_	452-86-8	HMDB0000873	YES
5	Oricinol	123.0454	C_7_H_8_O	504-15-4	HMDB0033629	YES
6	2-Methylphenol	107.0506	C_7_H_8_O	95-48-7	HMDB0002055	YES
7	3-Cresol	107.0506	C_8_H_8_O	108-39-4	HMDB0002048	YES
8	4-Vinylphenol	119.0502	C_7_H_8_O_4_S	2628-17-3	HMDB0001861	YES
9	4-Cresol sulfate	187.0072	C_6_H_6_O_4_S	91978-69-7	HMDB0011635	YES
10	Phenylsulfate	172.9916	C_7_H_6_O_4_	937-34-8	HMDB0060015	YES
11	2,3-Dihydroxybenzoic acid	153.0195	C_7_H_6_O_4_	303-38-8	HMDB0000397	YES
12	2,4-Dihydroxybenzoic acid	153.0195	C_7_H_6_O_4_	89-86-1	HMDB0001837	YES
13	2,6-Dihydroxybenzoic acid	153.0195	C_7_H_6_O_4_	303-07-1	HMDB0013676	YES
14	Gentisic acid	153.0195	C_8_H_7_NO_4_S	490-79-9	HMDB0000152	YES
15	Indoxyl sulfate	212.0025	C_9_H_8_O_4_	487-94-5	HMDB0000682	YES
16	Monomethyl phthalate	179.035	C_9_H_7_NO	4376-18-5	HMDB0001873	YES
17	Indole-3-carboxaldehyde	144.0455	C_14_H_28_O_2_	487-89-8	HMDB0000468	YES
18	12-Methyltridecanoic acid	227.2018	C_12_H_24_O_2_	2724-57-4	HMDB0031072	YES
19	Dodecanoic acid	199.1705	C_11_H_22_O_2_	143-07-7	HMDB0000638	YES
20	Undecanoic acid	185.1551	C_5_H_6_O_4_	112-37-8	HMDB0000930	YES
21	Citraconic acid	129.0193	C_5_H_6_O_4_	498-23-7	HMDB0000642	YES
22	Itaconic acid	129.0193	C_5_H_6_O_4_	97-65-4	HMDB0000053	YES
23	Mesaconic acid	129.0193	C_5_H_8_O_3_	498-24-8	HMDB0000706	YES
24	alpha-Ketoisovaleric acid	115.0401	C_6_H_10_O_3_	759-05-7	HMDB0000019	YES
25	Ketoleucine	129.0558	C_9_H_18_O_2_	816-66-0	HMDB0000695	YES
26	Pelargonic acid	157.1234	C_10_H_20_O_2_	112-05-0	HMDB0000891	YES
27	Capric acid	171.1396	C_8_H_16_O_2_	334-48-5	HMDB0000511	YES
28	Caprylic acid	143.1079	C_8_H_16_O_2_	124-07-2	HMDB0000482	YES
29	Valproic acid	143.1079	C_12_H_10_N_4_O_2_	99-66-1	HMDB0001877	YES
30	Lumichrome	241.0734	C_6_H_6_N_2_O	1086-80-2	HMDB0254199	YES
31	Nicotinamide	123.0553	C_10_H_14_N_2_	98-92-0	HMDB0001406	YES
32	Anabasine	163.123	C_10_H_14_N_2_	494-52-0	HMDB0034360	YES
33	Nicotine	163.123	C_6_H_12_O_2_	1954/11/5	HMDB0001934	YES
34	Caproic acid	115.0765	C_6_H_12_O_2_	142-62-1	HMDB0000535	YES
35	Isocaproic acid	115.0765	C_10_H_12_N_2_O	646-07-1	HMDB0000689	YES
36	N-Formylnornicotine	178.0976	C_7_H_8_N_2_O_2_	17770-06-6	HMDB0036484	YES
37	N1-Methyl-2-pyridone-5-carboxamide	153.0658	C_9_H_10_O_2_	701-44-0	HMDB0004193	YES
38	2′-Hydroxy-4′-methylacetophenone	151.0754	C_9_H_10_O_2_	876-02-0	-	YES
39	2′-Hydroxy-5′-methylacetophenone	151.0754	C_8_H_8_O_2_	1450-72-2	-	YES
40	2-Methylbenzoic acid	135.0451	C_9_H_10_O_3_	118-90-1	HMDB0001853	NO
41	2-Phenyllactic acid	165.0557	C_8_H_8_O_2_	828-01-3	HMDB0000779	NO
42	3-Hydroxyacetophenone	135.0451	C_7_H_6_O_2_	121-71-1	HMDB0001860	NO
43	3-hydroxybenzaldehyde	121.0295	C_9_H_8_O_2_	100-83-4	HMDB0001885	NO
44	3-Phenylpropanoic acid	149.061	C_9_H_10_O_2_	501-52-0	HMDB0000764	NO
45	4′-Hydroxy-3′-methylacetophenone	151.0754	C_8_H_8_O_2_	876-02-0	-	NO
46	4′-Hydroxyacetophenone	135.0451	C_9_H_8_O_2_	99-93-4	HMDB0001860	NO
47	4-Chromanone	147.0451	C_9_H_10_O_3_	491-37-2	HMDB0001926	NO
48	4-Ethoxybenzoic acid	165.0557	C_7_H_6_O_2_	619-86-3	HMDB0001874	NO
49	4-Hydroxybenzaldehyde	121.0295	C_7_H_8_O_2_	123-08-0	HMDB0001849	NO
50	4-Hydroxybenzyl alcohol	123.0451	C_8_H_8_O_2_	623-05-2	HMDB0001850	NO
51	4-Hydroxyphenylacetaldehyde	135.0451	C_8_H_8_O_2_	5703-23-9	HMDB0001851	NO
52	4-Methylbenzoic-acid	135.0451	C_9_H_10_O_2_	1999/4/7	HMDB0001852	NO
53	4′-Hydroxy-2′-methylacetophenone	151.0754	C_9_H_10_O_3_	876-02-0	-	NO
54	Ethyl 3-hydroxybenzoate	165.0557	C_9_H_10_O_3_	7781-98-8	HMDB0001875	NO
55	Ethyl 4-hydroxybenzoate	165.0557	C_10_H_10_O_4_	120-47-8	HMDB0001876	NO
56	Hydroferulic acid	193.0506	C_9_H_10_O_4_	1135-23-5	HMDB0002034	NO
57	Hydroxyphenyllactic acid	181.0506	C_8_H_8_O_2_	17114-20-0	HMDB0000755	NO
58	Phenylacetic acid	135.0451	C_11_H_15_N_5_O_3_S	103-82-2	HMDB0000209	NO
59	5′-Methylthioadenosine	298.0969	C_8_H_9_NO_3_	2457-80-9	HMDB0001173	NO
60	Pyridoxal (Vitamin B6)	168.0655	C_8_H_11_NO_3_	66-72-8	HMDB0001545	NO
61	Pyridoxine	170.0817	C_5_H_10_O_2_	65-23-6	HMDB0000239	NO
62	Isovaleric acid	101.0609	C_5_H_10_O_2_	503-74-2	HMDB0000718	NO
63	Valeric acid	101.0609	C_7_H_6_O_2_	109-52-4	HMDB0000892	NO
64	Benzoic acid	121.0296	C_6_H_12_O_3_	65-85-0	HMDB0001870	NO
65	2-Hydroxyhexanoic acid	131.0714	C_8_H_16_O_3_	6064-63-7	HMDB0001624	NO
66	3-Hydroxyoctanoic acid	159.1022	C_6_H_12_O_3_	14292-27-4	HMDB0000708	NO
67	Hydroxyisocaproic acid	131.0714	C_5_H_9_NO_3_	498-36-2	HMDB0000665	NO
68	Propionylglycine	131.0586	C_9_H_12_N_2_O_5_	14163-40-9	HMDB0000823	NO
69	2′-Deoxyuridine	227.0675	C_24_H_40_O_4_	951-78-0	HMDB0000012	NO
70	Deoxycholic acid	391.2855	C_24_H_40_O_4_	83-44-3	HMDB0000624	NO
71	Hyodeoxycholic acid	391.2855	C_24_H_40_O_4_	83-49-8	HMDB0000711	NO
72	Ursodeoxycholic acid	391.2855	C_27_H_43_NO_3_	128-13-2	HMDB0000946	NO
73	Isoverticine	430.3317	C_27_H_43_NO_3_	23496-41-5	-	NO
74	Peimine	430.3317	C_27_H_43_NO_3_	23496-41-5	HMDB0039269	NO
75	Peiminine	430.3317	C_9_H_9_NO_4_	18059-10-4	HMDB0039270	NO
76	Salicyluric acid	194.0461	C_11_H_11_NO_3_	487-54-7	HMDB0000840	NO
77	Indole-3-pyruvic acid	204.0669	C_5_H_10_O_3_	1821-52-9	HMDB0000671	NO
78	3-Hydroxyisovaleric acid	117.0558	C_7_H_7_NO_2_	4026-18-0	HMDB0000407	NO
79	4-Aminobenzoic acid	138.0549	C_5_H_4_N_4_O	150-13-0	HMDB0001392	NO
80	Hypoxanthine	137.0458	C_5_H_4_N_4_	68-94-0	HMDB0000157	NO
81	Purine	119.0365	C_10_H_7_NO_3_	120-73-0	HMDB0000626	NO
82	Kynurenic acid	190.0498	C_11_H_11_NO_3_	492-27-3	HMDB0000715	NO
83	N-Cinnamoylglycine	204.0669	C_9_H_9_NO_3_	16534-24-0	HMDB0011621	NO
84	Hippuric acid	178.051	C_10_H_11_NO_3_	495-69-2	HMDB0000714	NO
85	Phenylacetylglycine	194.0811	C_5_H_10_O_3_	500-98-1	HMDB0000821	NO
86	2-Methyl-3-hydroxybutyric acid	117.0559	C_14_H_18_N_2_O_2_	473-86-9	HMDB0000354	NO
87	Hypaphorine	247.144	C_6_H_5_NO_2_	487-58-1	HMDB0061115	NO
88	Isonicotinic acid	124.0392	C_6_H_5_NO_2_	55-22-1	HMDB0060665	NO
89	Nicotinate	124.0393	C_24_H_40_O_5_	59-67-6	HMDB0001488	NO
90	Allocholic acid	407.2807	C_24_H_40_O_5_	2464-18-8	HMDB0000505	NO
91	beta-Muricholic acid	407.2807	C_9_H_9_NO_4_	2393-46-2	HMDB0002538	NO
92	3-Hydroxyhippuric acid	194.0461	C_14_H_18_N_2_O_3_	1637-75-8	HMDB0006116	NO
93	Leucylphenylalanine	263.139	C_9_H_11_NO_2_	3918-92-1	HMDB0029134	NO
94	Phenylalanine	166.0862	C_11_H_12_N_2_O_2_	63-91-2	HMDB0000159	NO
95	Tryptophan	205.0977	C_6_H_11_NO_2_	73-22-3	HMDB0000929	NO
96	Hygric acid	130.0862	C_6_H_13_NO_2_	475-11-6	HMDB0094696	NO
97	Isoleucine	132.1018	C_6_H_13_NO_2_	73-32-5	HMDB0000172	NO
98	Leucine	132.1018	C_7_H_14_N_2_O_3_	61-90-5	HMDB0062203	NO
99	Theanine	174.0883	C_6_H_11_NO_3_	3081-61-6	HMDB0000464	NO
100	4-Acetamidobutyric acid	144.0667	C_6_H_6_N_2_O_2_	3025-96-5	HMDB0003681	NO
101	Urocanic acid	139.0501	C_5_H_11_NO_2_	104-98-3	HMDB0000301	NO
102	Valine	116.0718	C_9_H_9_NO_3_	72-18-4	HMDB0000883	NO
103	Desaminotyrosine	164.0717	C_9_H_11_NO_3_	501-94-0	HMDB0001174	NO
104	o-Tyrosine	182.081	C_9_H_11_NO_3_	587-29-1	HMDB0002228	NO
105	Tyrosine	182.081	C_6_H_12_N_2_O	60-18-4	HMDB0000158	NO
106	Pipecolamide	129.079	C_6_H_11_NO_2_	4038/10/8	HMDB0036692	NO
107	Pipecolic acid	130.0862	C_5_H_11_NO_2_	3105-95-1	HMDB0000716	NO
108	5-Aminopentanoic acid	116.0718	C_7_H_14_N_2_O_3_	660-88-8	HMDB0003322	NO
109	N2-Acetylornithine	173.093	C_4_H_9_NO_2_	3232/2/4	HMDB0003354	NO
110	2-Aminobutyric acid	102.0561	C_4_H_9_NO_2_	1492-24-6	HMDB0000452	NO
111	4-Aminobutyric acid (GABA)	102.0561	C_9_H_16_O_4_	1956/12/2	HMDB0000882	NO
112	Azelaic acid	187.0978	C_8_H_6_O_4_	123-99-9	HMDB0000784	NO
113	Terephthalic-Acid	165.0195	C_8_H_6_O_4_	100-21-0	HMDB0002428	NO
114	Phthalic acid	165.0195	C_8_H_6_O_4_	88-99-3	HMDB0000241	NO
115	gamma-Glutamylphenylalanine	295.129	C_5_H_9_NO_3_	7432-24-8	HMDB0000594	NO
116	5-Aminolevulinic acid	130.0511	C_5_H_6_O_5_	106-60-5	HMDB0001149	NO
117	alpha-Ketoglutaric acid (alpha-KG)	145.0144	C_7_H_11_N_3_O_2_	328-50-7	HMDB0000208	NO
118	1-Methylhistidine	170.0926	C_6_H_9_N_3_O_2_	332-25-6	HMDB0000001	NO
119	Histidine	156.0767	C_10_H_16_N_4_O_3_	71-00-1	HMDB0000177	NO
120	Anserine	241.1295	C_7_H_12_O_5_	584-85-0	HMDB0000194	NO
121	2-Isopropylmalic acid	161.0455	C_12_H_17_N_4_OS	3237-44-3	HMDB0000623	NO
122	Thiamine	265.1125	C_7_H_8_O_2_	59-43-8	HMDB0000235	NO
